# Therapeutically dosed low molecular weight heparins in renal impairment: a nationwide survey

**DOI:** 10.1007/s00228-022-03344-9

**Published:** 2022-06-17

**Authors:** Tessa Corrine Catherina Jaspers, A. Keyany, B. Maat, K. Meijer, P. M. L. A. van den Bemt, N. Khorsand

**Affiliations:** 1 Department of Hospital Pharmacy, Elisabeth TweeSteden Hospital, Tilburg, The Netherlands; 2 Department of Hematology, University Medical Center Groningen, Groningen, The Netherlands; 3 Department of Clinical Pharmacy and Pharmacology, University Medical Center Groningen, Groningen, The Netherlands; 4 Department of Hospital Pharmacy, OLVG, Amsterdam, The Netherlands

**Keywords:** “Heparin, Low molecular weight”, LMWH, Renal impairment, Thrombosis, Hemorrhage

## Abstract

**Purpose:**

International guidelines vary in their recommendations whether or not to reduce the therapeutic dose of low molecular weight heparins (LMWHs) in renal impairment. The use of anti-Xa monitoring as a basis of dose adjustments is also a matter of debate. As this may lead to variations in treatment policies, we aimed to study the treatment policies of therapeutically dosed LMWHs in renal impairment in Dutch hospitals.

**Methods:**

An 11-item survey was distributed between June 2020 and March 2021 to hospital pharmacists, representing Dutch hospital organisations. Primary outcomes were the dosing regimens of therapeutically dosed LMWHs in renally impaired patients. Secondary outcomes were the proportion of hospitals that used anti-Xa monitoring and the anti-Xa target range used.

**Results:**

There was a response from 56 of 69 (81%) Dutch hospital organisations where in each case a hospital pharmacist completed the survey. In these hospitals, 77 LMWH regimens were in use. In 76 of 77 (99%) regimens, a regular dose reduction was used at the start of treatment. Fifty-five of these hospitals used a dose reduction if estimated glomerular filtration rate (eGFR) < 50 ml/min and 17 used a dose reduction if eGFR < 30 ml/min. Anti-Xa levels were not routinely monitored in 40% of regimens, while 22% monitored anti-Xa if eGFR < 50 ml/min, 27% if eGFR < 30 ml/min and 10% in other eGFR cutoff values. Target ranges of 1.0–2.0 IU/ml (once daily) and 0.5/0.6–1.0 IU/ml (twice daily) were used in 69% of regimens that included monitoring of anti-Xa.

**Conclusion:**

Treatment policies show substantial diversity in therapeutically dosed LMWHs in renally impaired patients. The most commonly used treatment regimen was a regular dose reduction if eGFR is < 50 ml/min, without anti-Xa monitoring.

**Supplementary information:**

The online version contains supplementary material available at 10.1007/s00228-022-03344-9.

## Introduction

Low molecular weight heparins (LMWHs) are widely used for thromboprophylaxis and for the (initial) treatment of thrombo-embolic events [1–5]. As applies to all anticoagulants, LMWHs are high-risk drugs because of the dose-related risk of bleeding versus the risk of under treatment.

The use of LMWHs as thromboprophylaxis and as treatment for thrombo-embolic events in patients with a good renal function is well established. There is no need to monitor anti-factor Xa (anti-Xa) and/or activated partial thromboplastin time (APTT) [2, 3, 5]. As with most medicines, renally impaired patients were excluded from the LMWH registration studies [6–15]. The effectiveness and safety of therapeutically dosed LMWHs in these patients have not been established in prospective randomised studies. For this reason, various guidelines [1, 2, 4] recommend not to prescribe therapeutically dosed LMWHs in renally impaired patients. Unfractionated heparin (UFH) is still the preferred treatment in those patients according to most guidelines, mainly because its effect can be monitored by measuring the APTT [1, 2, 4]. However, multiple studies, although retrospective, have concluded that LMWHs are at least as effective and safe in renally impaired patients [17–20]. This, together with the ease of use of LMWHs, leads to their frequent use in renally impaired patients in the Netherlands.

Additionally, due to a lack of evidence, guidelines vary on their recommendations on LMWH dosing strategies and the usefulness of anti-Xa monitoring in renally impaired patients, as displayed in Table 1. Some guidelines [1–4, 16, 21–26] recommend a dose reduction, whereas others recommend not to use or give varying recommendations depending on the type of LMWH [5, 27–32]. Moreover, some guidelines recommend to apply anti-Xa-based dose adjustments [3, 4], whereas others recommend not to use or to be very cautious with anti-Xa-based dose adjustments due to a lack of evidence [2, 5, 16, 21, 22, 32].

**Table 1:** The varying guidance on dose reductions in renally impaired patients and anti-Xa monitoring

**Guideline**	**Recommendations on dose reductions**		**Recommendations on anti-Xa monitoring**			**Suggested anti-Xa range**
	**Reduce dose for ALL LMWHs**	**Reduce dose per LMWH**	**Monitor anti-Xa for ALL LMWHs**	**Monitor anti-Xa per LMWH**	**Consider Anti-Xa monitoring**	
BCSH 2006 [16]	< 30 ml/min^a^				< 30 ml/min	
ACCP 2012 [2]	< 30 ml/min^b^				< 30 ml/min^b^	Nadroparin: 0.6–1.0 IU/ml (BID), 1.3 (OD)^c^ Dalteparin: 1.05 IU/ml (OD) Enoxaparin: 0.6–1.0 IU/ml (BID) Tinzaparin: 0.85 IU/ml (OD)^c^
NfN 2012 [21, 22]	< 60 ml/min (75% if 30–60 ml/min 50% if < 30 ml/min)				< 60 ml/min	All LMWHs: BID: 0.6–1.0 IU/ml OD: 1.0–2.0 IU/ml
ISTH 2013 [4]	< 30 ml/min^d^		< 30 ml/min			
SIGN 2014 [3]	< 30 ml/min^a^		< 30 ml/min			
The Renal Drug Handbook [27]		Dalteparin: > 20 ml/min: no DR Enoxaparin: < 30 ml/min: DR to 1 mg/kg daily Tinzaparin: > 20 ml/min: no DR. < 20 ml/min: consider DR		Dalteparin: if eGFR < 30 ml/min		Dalteparin: 0.5–1.5 IU/ml
ASH 2018 [5]		Nadroparin: no DR [47] Dalteparin: no DR [48] Enoxaparin: < 30 ml/min: DR to 1 mg/kg daily [49] Tinzaparin: no DR [50]	Do not monitor anti-Xa			
ESC 2019 [1]	< 30 ml/min^a^					
NIV 2021 [21, 22]	< 60 ml/min (75% if 30–60 ml/min 50% if < 30 ml/min)				< 30 ml/min	
UpToDate [32]		Nadroparin: 30–50 ml/min: DR by 25–33%, < 30 ml/min: CI Dalteparin: no DR. < 30 ml/min: CI Enoxaparin: < 30 ml/min: DR Tinzaparin: no DR			< 30 ml/min	Nadroparin: 1.3 IU/ml^c^ Dalteparin: 0.5–1.5 IU/ml (OD) Enoxaparin: 0.6–1.0 IU/ml (BID) and > 1.0 IU/ml (OD) Tinzaparin: 0.85 IU/ml^c^
KNMP [23–26]	< 50 ml/min (75% if 30–50 ml/min 50% if < 30 ml/min)		< 30 ml/min			
Micromedex [28–31]		Nadroparin: no DR Dalteparin: no DR Enoxaparin: < 30 ml/min: DR to 1 mg/kg daily. 30–50 ml/min: DR to 0.8 mg/kg BID Tinzaparin: > 20 ml/min: no DR		Nadroparin: monitor anti-Xa (no eGFR cutoff) Dalteparin: if eGFR < 30 ml/min		Dalteparin: 0.5–1.5 IU/ml

*LMWH* low molecular weight heparin, *DR* dose reduction, *CI* contra-indicated, *OD* once daily, *BID* twice daily, *eGFR* estimated glomerular filtration rate, *ISTH* International Society of Thrombosis and haemostasis, *ASH* American Society of Haematology, *ESC* European Society of Cardiology, *ACCP* American College of Chest Physicians, *BCSH* British Society for Haematology, *SIGN* Scottish Intercollegiate Guidelines Network, *NfN* Dutch Federation of Nephrology, *NIV* Dutch Internists Association, *KNMP* Royal Dutch Pharmacists Association

^a^No details on DR are stated

^b^Guideline recommends to monitor anti-Xa and/or apply dose reduction of LMWH if LMWH is used in eGFR < 30 ml/min

^c^No range is stated

^d^Dose reduction based on anti-Xa levels in eGFR < 30 ml/min

Monitoring of anti-Xa levels may be used to monitor the effects of LMWH exposure, but such monitoring has many shortcomings [5, 33–35]. For example, a number of studies have failed to demonstrate a correlation between anti-Xa levels and LMWH effectiveness and/or bleeding risk [34–38]. Also, the only prospective randomised study on anti-Xa monitoring failed to demonstrate that anti-Xa-based dose adjustment leads to a larger proportion of anti-Xa levels within the range [33]. Moreover, these ranges are based on anti-Xa levels sampled 3–5 h after LMWH administration. In practice, the time of sampling is often outside this interval, which makes the corresponding anti-Xa level difficult to interpret [35, 39, 40]. Finally, the current target ranges are rarely achieved in both patients with normal renal function and in renal impairment [41–46].

In conclusion, the lack of clear evidence leads to conflicting recommendations on therapeutically dosed LMWHs in treatment guidelines. This may lead to varying hospital treatment policies for renally impaired patients. Therefore, we aimed to study the treatment policies of therapeutically dosed LMWHs in renal impairment in Dutch hospitals.

## Methods

### Design

This was a cross-sectional study using a survey among hospital pharmacists representing Dutch hospital organisations. The digital survey was distributed between June 2020 and March 2021. There was no incentive to fill out the survey and participation was voluntary.

### Setting

The Netherlands has 69 hospital organisations with hospital pharmacies, covering 114 different inpatient hospital locations [51]. In March 2021, these hospital organisations consisted of 8 university medical centres, 26 teaching hospitals and 35 general hospitals [51, 52]. Nadroparin, dalteparin, enoxaparin and tinzaparin are licenced LMWHs in the Netherlands.

### Survey

Data were collected using an 11-item survey in Word to identify the LMWH treatment policy for renally impaired patients in Dutch hospitals. The survey was developed by TJ and was reviewed by the other authors. The first item focused on the type(s) of LMWH(s) in use. Three items focused on the dosing regimen of LMWHs. The next three items focused on anti-Xa monitoring and anti-Xa-based dose adjustments. In addition, three items covered the hospital-wide treatment protocol. The last item requested additional comments. A copy of the survey is included in Supp. Appendix 1.

### Outcome measures

Primary outcomes were the different dosing regimens of therapeutically dosed LMWHs in renally impaired patients. Secondary outcomes were the proportion of Dutch hospitals that applied anti-Xa monitoring and the anti-Xa target ranges that were used.

### Data collection

Initially, the survey was distributed with the assistance of the Dutch Association of Hospital Pharmacists (NVZA) and the Special Interest Group Hematology of the NVZA. Primarily, the survey was distributed by the NVZA webpage, which is only accessible to NVZA members. NVZA members were automatically notified by e-mail. In this notification, hospital pharmacists with a focus on anticoagulation in their hospital were requested to complete the survey and to submit a copy of their hospital-wide treatment protocol, if available. If there was no response from a hospital organisation within 30 days, the hospital pharmacist with a focus on anticoagulation in these hospitals was personally contacted by phone or e-mail and asked to complete the survey. They received a second and third reminder by e-mail. Completed surveys and treatment protocols were returned by e-mail. The survey answers were checked against the treatment protocols, if available.

For each responder, the type of hospital, type(s) of LMWH in use and the availability of a hospital-wide treatment protocol were collected as general characteristics.

### Statistical analysis

Data analysis was performed with Excel (Microsoft Corporation, Redmond, USA). Results were analysed using descriptive statistics. Categorical variables were presented as percentages. Additional comments to questions and responses to open questions were grouped if they occurred ≥ 2 and then counted.

## Results

### Response rate and responder characteristics

For 56/69 (81%) hospital organisations, a hospital pharmacist completed the survey. For every hospital, only one hospital pharmacist responded.

The most commonly used LMWH for therapeutic indications was nadroparin (9.500 IE anti-Xa/ml), which was used in 32 (57%) of responding hospitals. Seven of these 32 hospitals also had the high nadroparin concentration (19.000 IE anti-Xa/ml) in use. Dalteparin was the second most frequently used LMWH in 21 (38%) hospitals, followed by tinzaparin and enoxaparin which was used in 14 hospitals (25%) and 10 hospitals (18%), respectively. Two or more LMWHs were used in 18 hospitals (nadroparin 9.500 IE/ml and nadroparin 19.000 IE/ml were considered to be one LMWH). In total, 77 LMWH regimens were in use.

Of the 56 hospitals that responded, 41 (73%) stated that they had a hospital-wide treatment protocol with dosing recommendations for therapeutically dosed LMWHs in renally impaired patients. Only twenty (49%) of these hospitals submitted their protocol. The majority of these guidelines were based on the Dutch guidelines “Antistolling met laagmoleculairgewicht heparines (LMWH) bij nierinsufficiëntie” of the Dutch Federation of Nephrology (NfN), “Behandeling VTE met LMWH bij nierfunctiestoornissen” of the Dutch Internists Association (NIV) and the database of the Koninklijke Nederlandse Maatschappij ter bevordering der Pharmacie (KNMP).

The characteristics of the hospital organisations are included in Table 2.

**Table 2:** Characteristics of the responders

**Hospital type**	**Responders,** ***n***	**Total n in the Netherlands**	**%**
-Academic hospitals	4	8	50
-Teaching hospitals	20	26	77
-Community hospitals	32	35	91
**LMWH(s) in use**		**Hospitals, *n* (% of hospitals)**	
-Nadroparin 9.500 IE anti-Xa/ml (Fraxiparine®)		32 (57)	
-Nadroparin 19.000 IE anti-Xa/ml (Fraxiparine Forte®)		7 (22)	
-Dalteparin (Fragmin^®^)		21 (38)	
-Enoxaparin (Clexane^®^, Inhixa®)		10 (18)	
-Tinzaparin (Innohep^®^)		14 (25)	
**Availability of a hospital-wide treatment protocol**		**Hospitals, *n* (% of hospitals)**	
-Yes		41 (73)	
-No		15 (27)	
**Hospital-wide treatment protocol is based on:**		**Hospitals, *n* (% of hospitals with protocol)**	
-Database of the KNMP (Royal Dutch Pharmacists Association)		**16 (39)**	
-Summary of Product Characteristics of the LMWH in use		6 (15)	
-Guideline ‘anticoagulation with LMWH in patients with renal impairment’ of the Dutch Federation of Nephrology		26 (63)	
-Publicly available protocols of other hospitals		2 (5)	
-Own experience/research with dose reductions and anti-Xa levels		3 (7)	
-Other		13 (32)	

*LMWH* low molecular weight heparin

### Dosing regimens in renally impaired patients

**Fig. 1:**
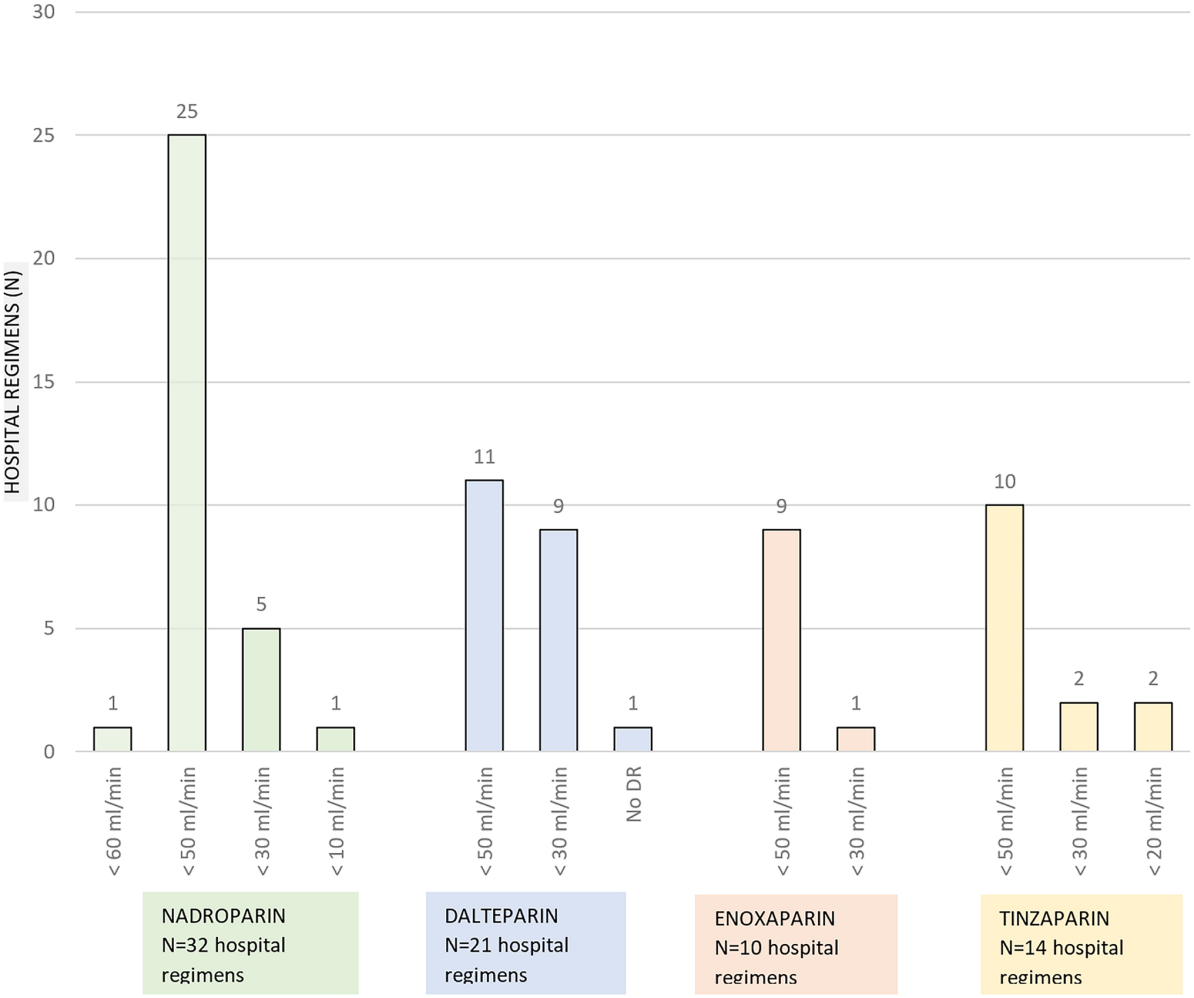
Dose reductions in renal impairment. The number of hospitals that did resp. did not apply LMHW dose reduction, categorised by LMWH type and by eGFR cutoff value. LMWH doses were reduced at certain eGFR cutoff values and below. DR, dose reduction. The number of hospital regimens exceeds the aforementioned 56 hospitals, as there are a number of hospitals that uses multiple LMWHs

The answers regarding dose reductions per LMWH are depicted in Fig. 1. In 76 of 77 (99%) LMWH regimens, a regular dose reduction of therapeutically dosed LMWHs was applied at the start of treatment. Only one hospital did not apply a dose reduction at the start of treatment. In 55 of 77 (71%) regimens, a dose reduction was applied in patients with an eGFR < 50 ml/min. Seventeen (22%) only applied a dose reduction in patients with an eGFR cutoff value of < 30 ml/min. One hospital responded that they did not apply a dose reduction for dalteparin in renally impaired patients. Four hospitals indicated other eGFR cutoff points for dose reductions.

There was variation in the degree of dose reduction and the eGFR at which it was applied; 50 (66%) applied a dose reduction of 25% in patients with eGFR 30–50 ml/min and a dose reduction of 50% in eGFR < 30 ml/min. In 8 of 76 (11%) LMWH regimens that applied dose reduction, no dose reduction was applied in eGFR 30–50 ml/min and a dose reduction of 50% in eGFR < 30 ml/min. In 18 regimens, other dosing regimens were applied.

In 63 of 76 (83%) LMWH regimens that applied dose reduction, a full dose was administered at the first dose, after which dose reduction was applied. In 7 of 76 (9%) LMWH regimens, the first dose was reduced in the same way as subsequent doses. For 6 regimens, this was unknown.

### Anti-Xa monitoring

**Fig. 2:**
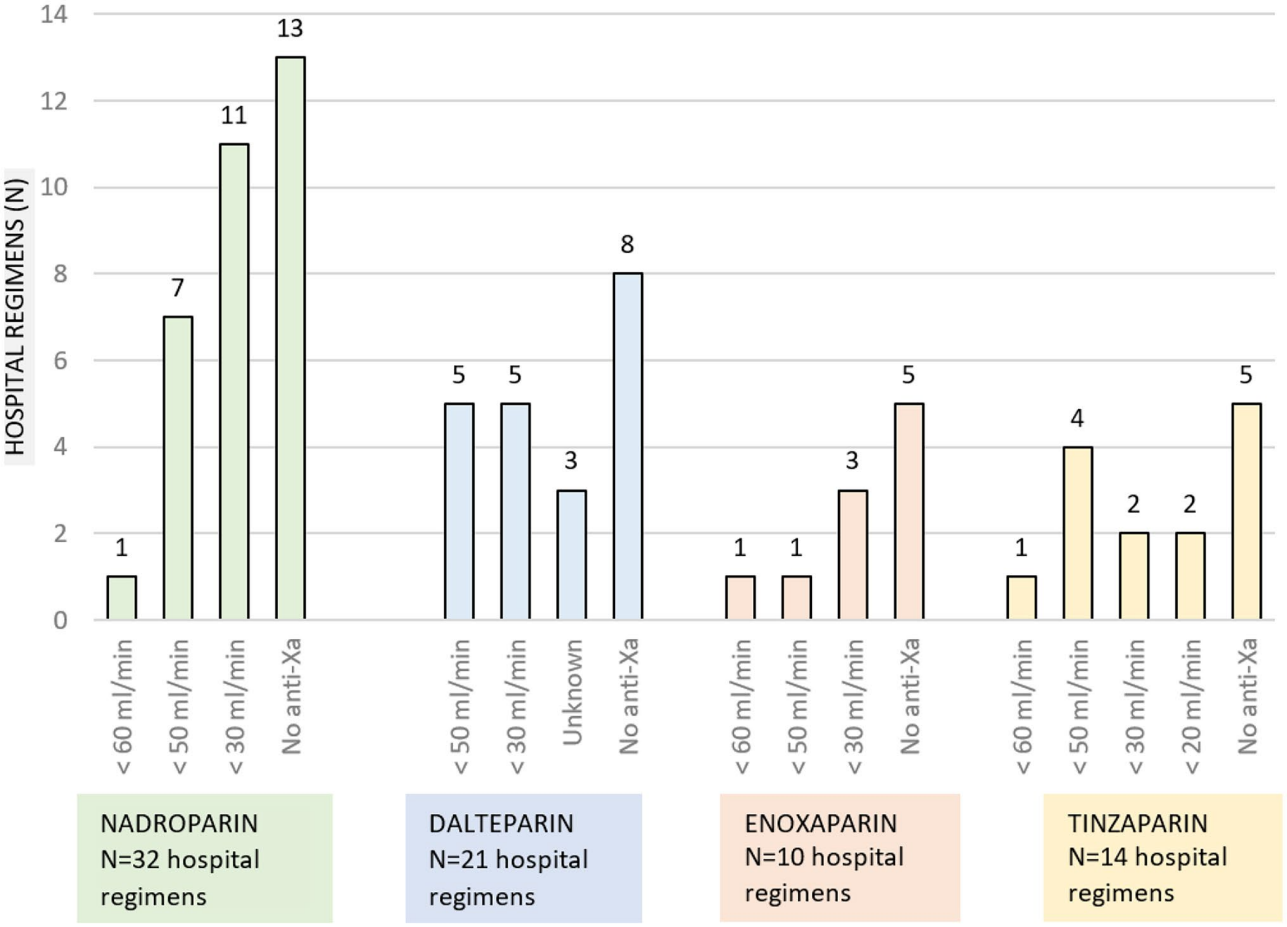
Anti-Xa monitoring in renal impairment. The number of hospital regimens that did or did not apply anti-Xa monitoring, categorised by LMWH type and by renal function. No anti-Xa, no anti-Xa monitoring. Unknown: Three hospitals reported that they monitor anti-Xa, but an eGFR cutoff value was not noted. The number of hospital regimens exceeds the aforementioned 56 hospitals, as there are a number of hospitals that use multiple LMWHs

When categorised per LMWH and split per reported eGFR cutoff for anti-Xa monitoring (Fig. 2), the most common treatment policy in renal impairment in all LMWHs was no anti-Xa monitoring (40%), followed by anti-Xa monitoring in eGFR < 30 ml/min (27%) and anti-Xa monitoring in eGFR < 50 ml/min (22%). Anti-Xa monitoring in eGFR < 60 ml/min (nadroparin and enoxaparin) and eGFR < 20 ml/min (tinzaparin) was rarely reported.

The anti-Xa target ranges per LMWH are depicted in Supp. Appendix 2. The target ranges of 1.0–2.0 IU/ml once daily (OD) and 0.6–1.0 IU/ml twice daily (BID) were used most frequently (*n* = 28, 52% of LMWH regimens with anti-Xa monitoring), followed by 1.0–2.0 IU/ml OD and 0.5–1.0 IU/ml BID (*n* = 9, 17%). The option “other,” that defined all different variants of target ranges, was reported for 31% (*n* = 17) of LMWH regimens that monitored anti-Xa.

The majority of responding hospitals (61%) applied dose adjustments based on anti-Xa levels that are below and above the target range. In the optional comment section, five hospitals reported that they never dose above 100% of the weight-based dose, while two hospitals reported that they occasionally largely exceed the maximum dose to keep anti-Xa levels within range.

### Overview treatment regimens per LMWH

**Fig. 3:**
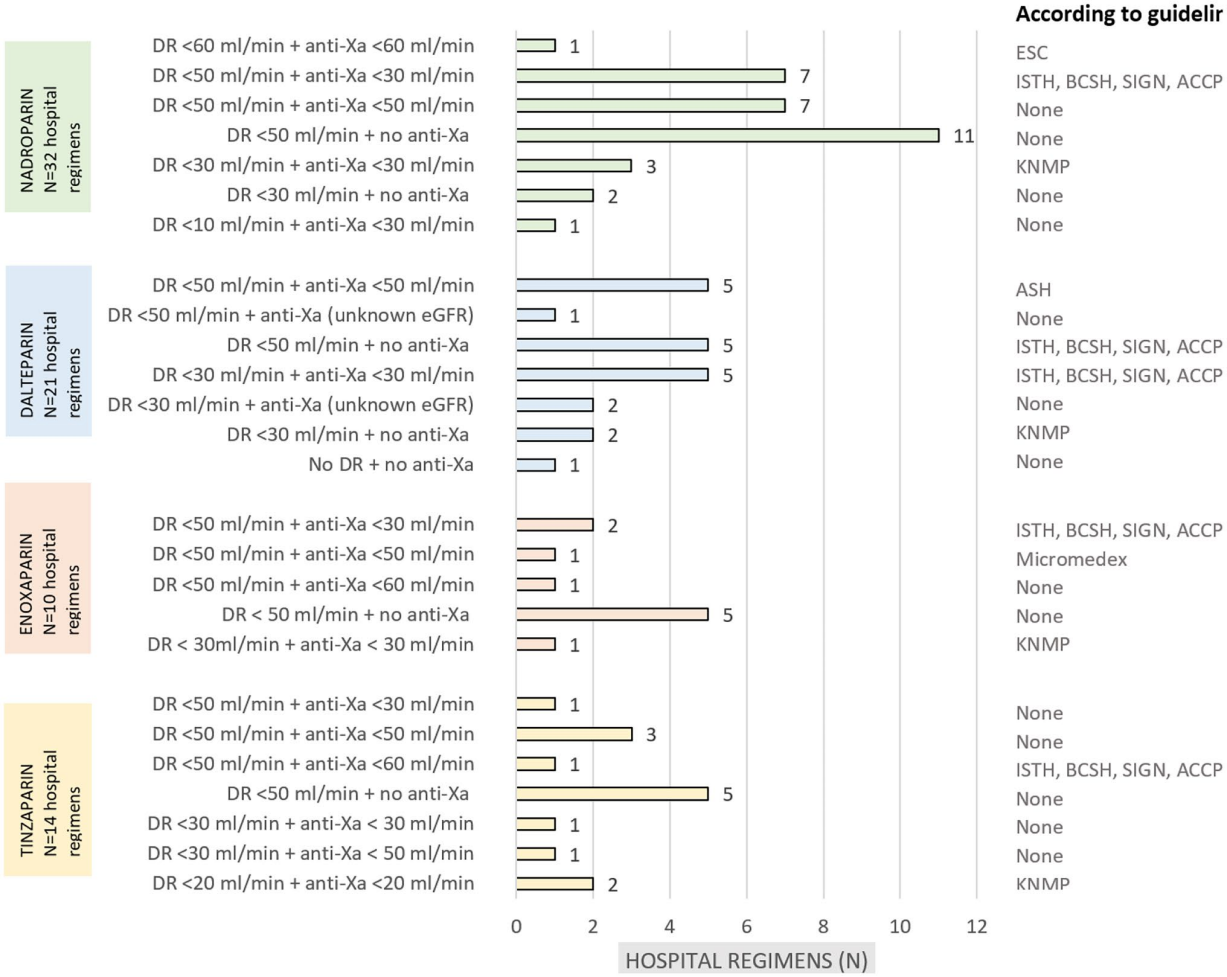
Overview treatment regimens per LMWH. An overview of the treatment regimens per LMWH is shown. DR, dose reduction; eGFR, estimated glomerular filtration rate; ISTH, International Society of Thrombosis and haemostasis; ASH, American Society of Haematology; ESC, European Society of Cardiology; ACCP, American College of Chest Physicians; BCSH, British Society for Haematology; SIGN, Scottish Intercollegiate Guidelines Network; NfN, Dutch Federation of Nephrology; NIV, Dutch Internists Association; KNMP, Royal Dutch Pharmacists Association * Three hospitals reported to monitor anti-Xa, but an eGFR cutoff value was not noted. The number of hospitals exceeds the aforementioned 56, as a number of hospitals use multiple LMWHs

An overview of the treatment regimens per LMWH is shown in Fig. 3. For all LMWHs, the most common treatment regimen was dose reduction in eGFR < 50 ml/min without anti-Xa monitoring (34%).

### Additional comments

Some responders had additional comments about the treatment policy in renal impairment. Six responders expressed their concerns whether the hospital protocol is generally followed in all wards. Also, four responders reported that anti-Xa levels are often incorrectly sampled, as they need to be sampled at peak anti-Xa level (3–5 h after LMWH administration).

## Discussion

We aimed to study the treatment policies of therapeutically dosed LMWHs in renally impaired patients in Dutch hospitals, by using a survey. For 56 of 69 (81%) Dutch hospital organisations, a hospital pharmacist completed the survey. Dose reduction appeared to be fairly uniform, as 76/77 (99%) of LMWH regimens applied a regular dose reduction at the start of LMWH treatment. In 64% of hospitals, a dose reduction of 25% was applied in eGFR 30–50 ml/min and a dose reduction of 50% in eGFR < 30 ml/min.

Anti-Xa levels were not routinely monitored in renally impaired patients in 40% of LMWH regimens. The target ranges of 1.0–2.0 IU/ml (once daily) and 0.5/0.6–1.0 IU/ml (twice daily) were used most frequently in all LMWHs (69%). Overall, dose reductions in eGFR < 50 ml/min without anti-Xa monitoring is the most common treatment regimen in Dutch hospitals, regardless of the type of LMWH.

Most guidelines recommend dose reduction in renally impaired patients, which is reflected in the fact that the majority of Dutch hospitals apply a dose reduction [1, 2, 16, 21, 32]. However, the dosing regimens vary greatly in these guidelines, which is also reflected in the dosing regimens applied in the Dutch hospitals. The majority of hospitals apply dose reductions as stated by the NfN, NIV and KNMP, as they recommend a 25% dose reduction in eGFR < 50–60 ml/min and 50% in eGFR < 30 ml/min [21, 23–26].

The most commonly used treatment regimen in Dutch hospitals is dose reduction in eGFR < 50 ml/min without anti-Xa monitoring for all types of LMWH (Fig. 3). This is surprising because this is not recommended in any of the guidelines (Table 1). Although the European Society of Cardiology guideline does not mention anti-Xa monitoring and American Society of Hematology is the only guideline that actively recommends not to monitor anti-Xa, both guidelines recommend a regular dose reduction in eGFR < 30 ml/min in most LMWHs, in contrast to the most common eGFR reduction point of < 50 ml/min in Dutch hospitals. The majority of guidelines recommends to monitor anti-Xa (in eGFR < 30 ml/min or < 60 ml/min), despite the many shortcomings mentioned in the same guidelines [2, 16, 21, 22, 32]. Although anti-Xa monitoring is also recommended in the Dutch guidelines of NfN, NIV and KNMP, which were mostly followed, we conclude that 40% of Dutch hospitals do not monitor anti-Xa levels in renally impaired patients [21–26].

A possible explanation for the lack of anti-Xa monitoring is a lack of clear evidence for its usefulness and its target levels, as well as the need for a simple treatment policy in daily practice. Moreover, the target values in the Dutch guidelines of 0.5/0.6–1.0 IU/ml and 1.0–2.0 IU/ml for a OD and BID regimen respectively are not evidence based [21, 22]. These data have been extrapolated from enoxaparin, because data on the other LMWHs are lacking [21, 22]. On theoretical grounds, different target ranges would be expected, because the LMWHs have different molecular weights, causing a different affinity for factor IIa and Xa [53, 54]. Moreover, there is a growing evidence that the stated target ranges are hardly achieved in the majority of patients regardless of renal function [35, 41, 43–46].

The response rate of 81% is high and all types of hospitals are represented. Notwithstanding these strengths, several limitations are present as well. First, of the 18 hospitals that used two or more LMWHs, three reported to use different treatment regimens for the different types of LMWH in use. Six of the remaining 15 hospitals did not describe a different treatment regimen for different LMWHs in the provided treatment protocol. It is unclear whether the other nine hospitals use the same treatment regimen for different LMWHs. We assumed a similar regimen as three hospitals did not encounter difficulties to report different treatment regimens in the survey and a similar treatment regimen was described in 6/15 hospitals that submitted a treatment protocol.

Moreover, it is possible that hospital pharmacists are not aware of the treatment policy that is applied in clinical practice. Generally, in the Netherlands, at least one hospital pharmacist per hospital focuses on anticoagulation. These hospital pharmacists are often involved in writing hospital-wide treatment protocols, are members of multidisciplinary antithrombotic committees and are consulted on issues relating to dosing and monitoring of LMWHs. We therefore considered the hospital pharmacist the primary source for hospital-wide treatment policy. A possible limitation related to the design of the study is that the assumption was made that the responders answered accurately and truthfully. For 20 hospitals, the survey answers could be validated against treatment protocols, as only 20 protocols had been submitted. However, no discrepancies were found between survey answers and treatment protocols, indicating an overall accurate response of hospital pharmacists.

A final limitation is that the study was only conducted in the Netherlands. A possible difference is that in other countries UFH is used more often instead of LMWHs in patients with eGFR < 30 ml/min, as they do not use the Dutch NfN and NIV guidelines [21, 22].

The most commonly used treatment regimen in Dutch hospitals is to apply a dose reduction in eGFR < 50 ml/min without anti-Xa monitoring, regardless of the type of LMWH. The effectiveness and safety of this treatment policy need to be confirmed in future research.

## Conclusion

This descriptive, cross-sectional study demonstrates substantial diversity in the treatment policy of therapeutically dosed LMWHs in renally impaired patients in Dutch hospitals. The most common treatment regimen is to apply a dose reduction in eGFR < 50 ml/min without anti-Xa monitoring.

## Supplementary information

Below is the link to the electronic supplementary material.

Supplementary file1 (DOCX 103 KB)

## References

[1] Konstantinides SV, Meyer G, Becattini C, Bueno H, Geersing GJ, Harjola VP et al (2019) ESC Guidelines for the diagnosis and management of acute pulmonary embolism developed in collaboration with the European Respiratory Society (ERS): The Task Force for the diagnosis and management of acute pulmonary embolism of the European Society of Cardiology (ESC). Eur Respir J 54(3) 10.1183/13993003.01647-2019 31473594

[2] Garcia DA, Baglin TP, Weitz JI, Samama MM (2012). Parenteral anticoagulants: antithrombotic therapy and prevention of thrombosis, 9th ed: American College of Chest Physicians Evidence-Based Clinical Practice Guidelines. Chest.

[3] Scottish Intercollegiate Guidelines Network (SIGN) (2010) Prevention and management of venous thromboembolism Edinburgh: SIGN (SIGN publication no. 122)

[4] Farge D, Debourdeau P, Beckers M, Baglin C, Bauersachs RM, Brenner B (2013). International clinical practice guidelines for the treatment and prophylaxis of venous thromboembolism in patients with cancer. J Thromb Haemost.

[5] Witt DM, Nieuwlaat R, Clark NP, Ansell J, Holbrook A, Skov J (2018). American Society of Hematology 2018 guidelines for management of venous thromboembolism: optimal management of anticoagulation therapy. Blood Adv.

[6] Blazing MA, de Lemos JA, White HD, Fox KA, Verheugt FW, Ardissino D (2004). Safety and efficacy of enoxaparin vs unfractionated heparin in patients with non-ST-segment elevation acute coronary syndromes who receive tirofiban and aspirin: a randomized controlled trial. JAMA.

[7] Cohen M, Blaber R, Demers C, Gurfinkel EP, Langer A, Fromell G (1997). The essence trial: efficacy and safety of subcutaneous enoxaparin in unstable angina and non-Q-wave MI: a double-blind, randomized, parallel-group, multicenter study comparing enoxaparin and intravenous unfractionated heparin: methods and design. J Thromb Thrombolysis.

[8] Daskalopoulos ME, Daskalopoulou SS, Tzortzis E, Sfiridis P, Nikolaou A, Dimitroulis D (2005). Long-term treatment of deep venous thrombosis with a low molecular weight heparin (tinzaparin): a prospective randomized trial. Eur J Vasc Endovasc Surg.

[9] Hull RD, Raskob GE, Brant RF, Pineo GF, Elliott G, Stein PD et al (2000) Low-molecular-weight heparin vs heparin in the treatment of patients with pulmonary embolism. American-Canadian Thrombosis Study Group. Arch Intern Med 160(2):229–36 10.1001/archinte.160.2.229 10647762

[10] Jolly SS, Faxon DP, Fox KA, Afzal R, Boden WE, Widimsky P (2009). Efficacy and safety of fondaparinux versus enoxaparin in patients with acute coronary syndromes treated with glycoprotein IIb/IIIa inhibitors or thienopyridines: results from the OASIS 5 (Fifth Organization to Assess Strategies in Ischemic Syndromes) trial. J Am Coll Cardiol.

[11] Karthaus M, Kretzschmar A, Kröning H, Biakhov M, Irwin D, Marschner N (2006). Dalteparin for prevention of catheter-related complications in cancer patients with central venous catheters: final results of a double-blind, placebo-controlled phase III trial. Ann Oncol.

[12] Luomanmäki K, Grankvist S, Hallert C, Jauro I, Ketola K, Kim HC (1996). A multicentre comparison of once-daily subcutaneous dalteparin (low molecular weight heparin) and continuous intravenous heparin in the treatment of deep vein thrombosis. J Intern Med.

[13] Ramacciotti E, Araújo GR, Lastoria S, Maffei FH, Karaoglan de Moura L, Michaelis W et al (2004) An open-label, comparative study of the efficacy and safety of once-daily dose of enoxaparin versus unfractionated heparin in the treatment of proximal lower limb deep-vein thrombosis. Thromb Res 114(3):149–53 10.1016/j.thromres.2004.05.009 15342210

[14] Sabatine MS, Morrow DA, Dalby A, Pfisterer M, Duris T, Lopez-Sendon J (2007). Efficacy and safety of enoxaparin versus unfractionated heparin in patients with ST-segment elevation myocardial infarction also treated with clopidogrel. J Am Coll Cardiol.

[15] White HD, Kleiman NS, Mahaffey KW, Lokhnygina Y, Pieper KS, Chiswell K (2006). Efficacy and safety of enoxaparin compared with unfractionated heparin in high-risk patients with non-ST-segment elevation acute coronary syndrome undergoing percutaneous coronary intervention in the Superior Yield of the New Strategy of Enoxaparin, Revascularization and Glycoprotein IIb/IIIa Inhibitors (SYNERGY) trial. Am Heart J.

[16] Baglin T, Barrowcliffe TW, Cohen A, Greaves M (2006). Guidelines on the use and monitoring of heparin. Br J Haematol.

[17] Lim W, Dentali F, Eikelboom JW, Crowther MA (2006). Meta-analysis: low-molecular-weight heparin and bleeding in patients with severe renal insufficiency. Ann Intern Med.

[18] Park D, Southern W, Calvo M, Kushnir M, Solorzano C, Sinnet M (2016). Treatment with dalteparin is associated with a lower risk of bleeding compared to treatment with unfractionated heparin in patients with renal insufficiency. J Gen Intern Med.

[19] Trujillo-Santos J, Schellong S, Falga C, Zorrilla V, Gallego P, Barrón M (2013). Low-molecular-weight or unfractionated heparin in venous thromboembolism: the influence of renal function. Am J Med.

[20] Crowther M, Lim W (2016). Use of low molecular weight heparins in patients with renal failure; time to re-evaluate our preconceptions. J Gen Intern Med.

[21] Nederlandse federatie van Nefrologie (NfN) (2012) Richtlijn Antistolling met laagmoleculairgewicht heparines (LMWH) bij nierinsufficiëntie [in Dutch]. Nieuwegein, the Netherlands: NfN

[22] Dutch Internists Association (NVI) (2021) Richtlijn antitrombotisch beleid. Behandeling LMWH bij nierfunctiestoornissen en risico op VTE [in Dutch]. Utrecht: Dutch Internists Association

[23] Royal Dutch Pharmacists Assciation (KNMP) (2021) DALTEPARINE NIERFUNCTIE. KNMP. [cited 2021–06–28]. Available from: https://kennisbank.knmp.nl

[24] Royal Dutch Pharmacists Assciation (KNMP) (2021) NADROPARINE NIERFUNCTIE. KNMP [cited 2021–06–28]. Available from: https://kennisbank.knmp.nl

[25] Royal Dutch Pharmacists Assciation (KNMP) (2021) ENOXAPARIN NIERFUNCTIE. KNMP [cited 2021–06–28]. Available from: https://kennisbank.knmp.nl

[26] Royal Dutch Pharmacists Assciation (KNMP) (2021) TINZAPARINE NIERFUNCTIE. KNMP [cited 2021–06–28]. Available from: https://kennisbank.knmp.nl

[27] Ashley C, Dunleavy A (2014) The renal drug handbook. 4 ed. London: CRC Press 276, 367, 1002

[28] IBM Micromedex (2021) Enoxaparin sodium. In: Dose adjustments. IBM Corporation. [cited 2021–06–28]. Available from: www.micromedexsolutions.com

[29] IBM Micromedex (2021) Nadroparin. In: Dose adjustments. IBM Corporation. [cited 2021–06–28]. Available from: https://www.micromedexsolutions.com

[30] IBM Micromedex (2021) Dalteparin sodium. In: Dose adjustments. IBM Corporation. [cited 2021–06–28]. Available from: https://www.micromedexsolutions.com

[31] IBM Micromedex (2021) Tinzaparin Sodium. In: Dose adjustments. IBM Corporation. [cited 2021–06–28]. Available from: https://www.micromedexsolutions.com

[32] Hull RD (2021) Suggested dose adjustments of low molecular weight (LMW) heparins in adults with renal insufficiency. Waltham, MA: UpToDate [cited 2021–02–25]. Available from: https://www.uptodate.com

[33] Alhenc-Gelas M, Jestin-Le Guernic C, Vitoux JF, Kher A, Aiach M, Fiessinger JN (1994). Adjusted versus fixed doses of the low-molecular-weight heparin fragmin in the treatment of deep vein thrombosis. Fragmin-Study Group Thromb Haemost.

[34] Bara L, Leizorovicz A, Picolet H, Samama M (1992). Correlation between anti-Xa and occurrence of thrombosis and haemorrhage in post-surgical patients treated with either Logiparin (LMWH) or unfractionated heparin. Post-surgery Logiparin Study Group Thromb Res.

[35] Hornung P, Khairoun M, Dekker FW, Kaasjager KAH, Huisman A, Jakulj L (2020). Dosage reduction of low weight heparin in patients with renal dysfunction: effects on anti-Xa levels and clinical outcomes. PLoS ONE.

[36] Falgá C, Capdevila JA, Soler S, Rabuñal R, Sánchez Muñoz-Torrero JF, Gallego P et al (2007) Clinical outcome of patients with venous thromboembolism and renal insufficiency. Findings from the RIETE registry. Thromb Haemost 98(4):771–6 17938800

[37] Prandoni P, Lensing AW, Büller HR, Carta M, Cogo A, Vigo M (1992). Comparison of subcutaneous low-molecular-weight heparin with intravenous standard heparin in proximal deep-vein thrombosis. Lancet.

[38] Walenga JM, Hoppensteadt D, Fareed J (1991). Laboratory monitoring of the clinical effects of low molecular weight heparins. Thromb Res Suppl.

[39] Lin A, Vazquez SR, Jones AE, Witt DM (2019). Description of anti-Xa monitoring practices during low molecular weight heparin use. J Thromb Thrombolysis.

[40] Thomas O, Lybeck E, Strandberg K, Tynngård N, Schött U (2015). Monitoring low molecular weight heparins at therapeutic levels: dose-responses of, and correlations and differences between aPTT, anti-factor Xa and thrombin generation assays. PLoS ONE.

[41] Olie RH, Meertens NEL, Henskens YMC, Ten Cate H (2017). Empirically reduced dosages of tinzaparin in patients with moderate-to-severe renal insufficiency lead to inadequate anti-Xa levels. Nephron.

[42] Russcher M, Josephus Jitta N, Kraaijenhagen RJ, Fijnheer R, Pasker-de Jong PC, Gaillard CA (2013). Preemptive dosage reduction of nadroparin in patients with renal failure: a retrospective case series. Clin Kidney J.

[43] Shprecher AR, Cheng-Lai A, Madsen EM, Cohen HW, Sinnett MJ, Wong ST (2005). Peak antifactor xa activity produced by dalteparin treatment in patients with renal impairment compared with controls. Pharmacotherapy.

[44] Smit R, Marum RJ Van, Péquériaux NCV, Hollander AAMJ, Bleeker MWP, Hermens WAJJ et al (2016) Prevalentie van correcte anti-Xa-bloedspiegels bij patiënten met verminderde nierfunctie op basis van dosisadvies conform richtlijn Nederlandse Federatie voor Nefrologie. Dutch Platform for Pharmaceutical Research (NPFO). [cited 2021–06–28]

[45] van Bergen EDP, Huisman A, Welsing PMJ, de Winter MA, Rookmaaker MB, Kaasjager HAH (2019). Dalteparin and anti-Xa: a complex interplay of therapeutic drug monitoring. Neth J Med.

[46] Ojik AL van, Hemmelder M, Hoogendoorn M, Folkeringa R, Smit R, Derijks HJ et al (2016) Anti-Xa-activiteit van therapeutisch nadroparine bij verminderde nierfunctie en behandeling conform richtlijn Nederlandse federatie voor Nefrologie: vergelijking met standaarddosis bij normale nierfunctie. Dutch Platform for Pharmaceutical Research (NPFO). [cited 2021–06–28]

[47] Mylan Healthcare BV (2021) Fraxiparine 9.500IE/1ml oplossing voor injectie. Samenvatting van de productkenmerken. [cited 2022–03–03]. Available from: geneesmiddeleninformatiebank.nl

[48] Pfizer (2015) Fragmin oplossing voor injectie. Samenvatting van de productkenmerken. [cited 2022–03–03]. Available from: geneesmiddeleninformatiebank.nl

[49] Techdow Pharma Netherlands BV (2021) Inhixa 2.000 IE (20 mg)/0,2 ml oplossing voor injectie in een voorgevulde spuit. Samenvatting van de productkenmerken. [cited 2022–03–03]. Available from: geneesmiddeleninformatiebank.nl

[50] Leo Pharma BV (2019) Innohep oplossing voor injectie. Samenvatting van de productkenmerken. [cited 2022–03–03]. Available from: geneesmiddeleninformatiebank.nl

[51] National Institute for Public Health and the Environment (RIVM) (2021) Algemene en academische ziekenhuizen. In: Locaties. In: Regionaal & Internationaal. In: Ziekenhuiszorg. RIVM. [cited 2021–06–28]. Available from: https://www.volksgezondheidenzorg.info/onderwerp/ziekenhuiszorg/regionaal-internationaal/locaties#node-algemene-en-academische-ziekenhuizen

[52] Cooperating Top Clinical Training hospitals (STZ) (2021) Het STZ topklinisch zorgregister. Bureau STZ. [cited 2021–06–28]. Available from: https://www.stz.nl/30160/topklinisch-zorgregister

[53] Johansen KB, Balchen T (2013). Tinzaparin and other low-molecular-weight heparins: what is the evidence for differential dependence on renal clearance?. Exp Hematol Oncol.

[54] Hetzel GR, Sucker C (2005). The heparins: all a nephrologist should know. Nephrol Dial Transplant.

