# Supplementary material for: Therapeutically dosed low molecular weight heparins in renal impairment: a nationwide survey
Source: Eur J Clin Pharmacol. 2022 Jun 17;78(9):1469–79. doi: 10.1007/s00228-022-03344-9 (PMC9365729; doi:10.1007/s00228-022-03344-9)
Supplement: Supplementary file 1 — Supplementary file1 (DOCX 103 KB) [file 228_2022_3344_MOESM1_ESM.docx]

# Supplementary Appendix

## Survey questions

1. Which LMWH is used in therapeutic doses in your hospital? *(multiple options possible)*

Nadroparin (Fraxiparin®)

Nadroparin (Fraxiparin Forte®, Fraxodi®)

Enoxaparin (Clexane®, Lovenox®, Klexane®)

Dalteparin (Fragmin®, Fragmine®)

Tinzaparin (Innohep®)

|  |
| --- |

Other:

1. According to hospital policy, is dose reduction of therapeutically dosed LMWHs applied in renally impaired patients at the start of treatment?

Yes, if eGFR is < 50 ml/min

Yes, if eGFR is < 30 ml/min

Yes, if ______________________________________________________________

No, *proceed to question 6*

Not applicable, *proceed to question 6*

1. Is a dose reduction applied at the **first administered dose**?

No, a full dose is administered as the first dose

Yes, the first dose is reduced by _______%

Other: __________________________________________________________­­­____

1. How are **subsequent doses** adjusted?

25% dose reduction if eGFR is 30-50 ml/min and 50% dose reduction if eGFR is < 30 ml/min

No dose reduction if eGFR is 30-50 ml/min and 50% dose reduction if eGFR is < 30 ml/min

No dose reduction in advance. Dose adjustments are optionally done based on anti-Xa levels

Other: ______________________________________________________________

1. Is the anti-Xa activity routinely determined in renally impaired patients with an intended treatment duration of >3 days?

    Yes, in all patients with eGFR <______ml/min
    Yes, if the estimated treatment period is ≥_____days and eGFR is <______ml/min
    No, *proceed to question 10*

Other: ______________________________________________________________

1. What are the target values for the anti-Xa activity in your hospital for therapeutically dosed LMWHs?

| LMWH once daily dose: | …………-.........U/ml …………….. (number) hours after last dose |
| --- | --- |
| LMWH twice daily dose: | …………-……….U/ml …………..... (number) hours after last dose |
| Remarks: | _________________________________________________________ |

1. Are LMWH doses adjusted based on the anti-Xa activity?

|  | Yes, if the anti-Xa activity is too low or too high |
| --- | --- |
|  | Yes, *only* if the anti-Xa activity is **too high** |
|  | Yes, *only* if the anti-Xa activity is **too low** |
|  | Other: ______________________________________________________________ |
|  | No |

1. In your hospital, is there a protocol with dosing recommendations for therapeutically dosed LMWHs in renally impaired patients?

Yes, *proceed to question 9*.

No, *proceed to question 10*.

1. If yes, which literature and/or guidelines are these recommendations based on? *(multiple options possible)*

Database of the KNMP (Royal Dutch Pharmacists Association)

Summary of Product Characteristics of the LMWH in use

Guideline ‘anticoagulation with LMWH in patients with renal insufficiency’ of the Dutch Federation of Nephrology (NfN)

Other guidelines or clinical research. The following literature has been consulted: ________________________________________________________________

Other: __________________________________________________________

1. Is the hospital’s dosing guideline generally supported and used by all medical staff?

Yes

No

Other: _________________________________________________________

1. Do you have any comments or additional information?

____________________________________________________________________________

____________________________________________________________________________

____________________________________________________________________________

## 2. Anti-Xa target ranges per LMWH.


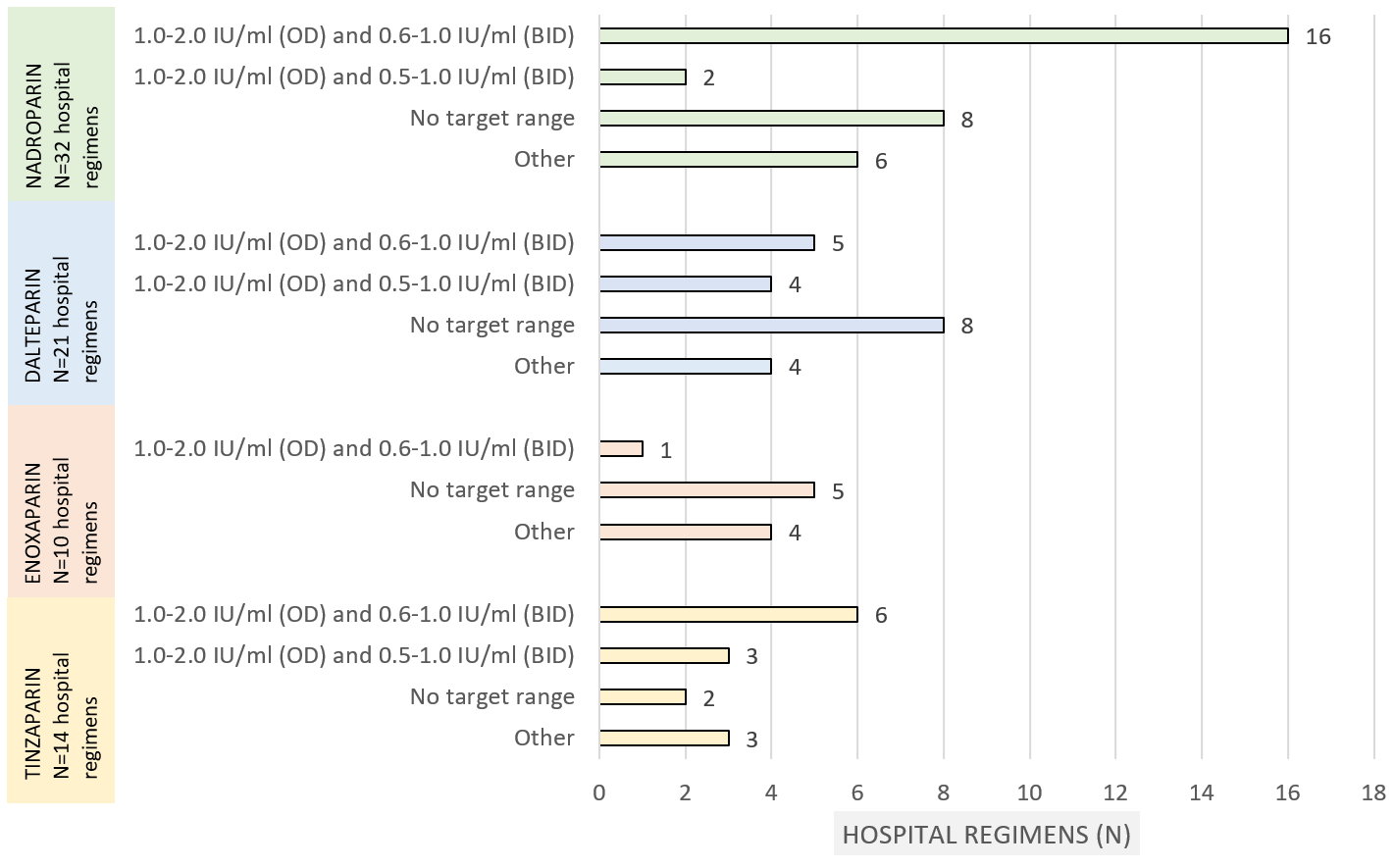


*The different anti-Xa target ranges, categorized per LMWH type are shown. The number of hospital regimens that used the stated target ranges are also depicted. IU: international units, OD: once daily, BID: twice daily. No target range – the hospital regimens in which no anti-Xa monitoring was described. Other – all target ranges that were reported only once, were grouped by this option. The number of hospital regimens exceeds the aforementioned 56 hospitals, as there is a number of hospitals that use multiple LMWHs.*
